# Genome-wide mapping of gene–microbiota interactions in susceptibility to autoimmune skin blistering

**DOI:** 10.1038/ncomms3462

**Published:** 2013-09-17

**Authors:** Girish Srinivas, Steffen Möller, Jun Wang, Sven Künzel, Detlef Zillikens, John F. Baines, Saleh M. Ibrahim

**Affiliations:** 1 Max Planck Institute for Evolutionary Biology, August-Thienemann-Street 2, D-24306 Plön, Germany; 2 Department of Dermatology, University of Lübeck, Ratzeburger Allee 160, D-23538 Lübeck, Germany; 3 Institute for Experimental Medicine, Christian-Albrechts-University of Kiel, Arnold-Heller-Street 3, D-24105 Kiel, Germany; 4 These authors contributed equally to this work

## Abstract

Susceptibility to chronic inflammatory diseases is determined by immunogenetic and environmental risk factors. Resident microbial communities often differ between healthy and diseased states, but whether these differences are of primary aetiological importance or secondary to the altered inflammatory environment remains largely unknown. Here we provide evidence for host gene–microbiota interactions contributing to disease risk in a mouse model of epidermolysis bullosa acquisita, an autoantibody-induced inflammatory skin disease. Using an advanced intercross, we identify genetic loci contributing to skin microbiota variability, susceptibility to skin blistering and their overlap. Furthermore, by treating bacterial species abundances as covariates with disease we reveal a novel disease locus. The majority of the identified covariate taxa are characterized by reduced abundance being associated with increased disease risk, providing evidence of a primary role in protection from disease. Further characterization of these putative probiotic species or species assemblages offers promising potential for preventative and therapeutic treatment development.

## Abstract (web summary)

The pathogenesis of inflammatory disorders afflicting the skin is multifactorial. Srinivas *et al*. show that diversity of the skin microbiota is a critical factor determining the susceptibility to epidermolysis bullosa acquisita, a chronic mucocutaneous autoimmune skin blistering disease.

## Graphical abstract

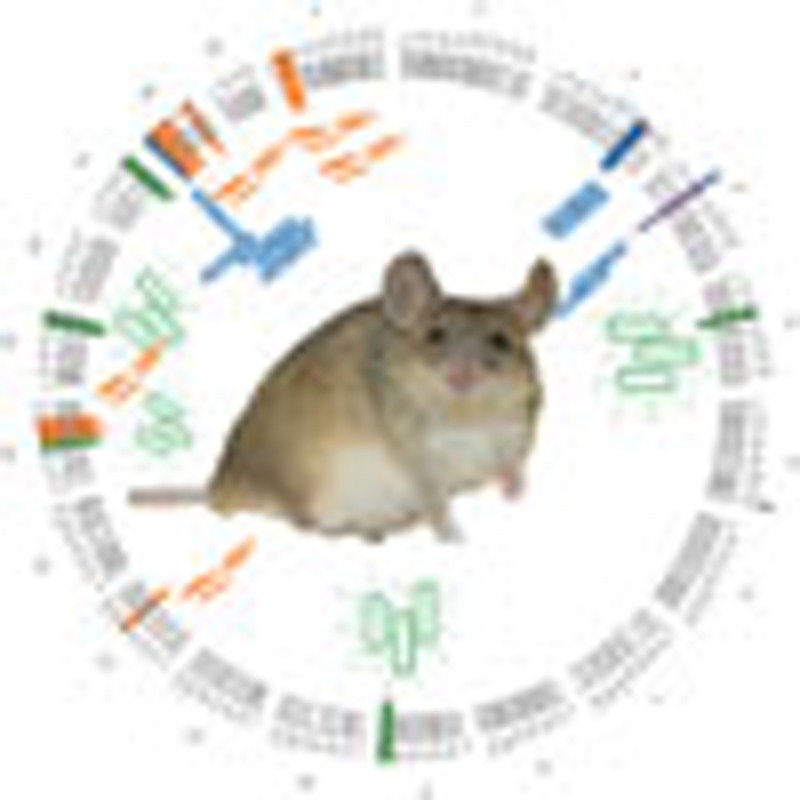

Diverse communities of symbiotic bacteria inhabit nearly every surface of the body exposed to the environment. The skin in particular is in constant contact with the environment and serves a critical barrier function, yet provides a range of niches to inhabiting microbial communities. A multitude of interactions between the skin microbiota, host and environment contribute to community structure over space and time and its potential contribution to changes in health status^1^,^2^. Recent landmark studies of the mouse gut microbiota using a quantitative trait locus (QTL) mapping approach unequivocally demonstrate the role of host genetics in shaping diversity between individuals^3^,^4^. Likewise, inflammatory disorders afflicting the skin such as psoriasis and atopic dermatitis harbour clear immunogenetic components, but whether these associations may be mediated by alterations in microbial community structure is unknown^1^,^5^.

Epidermolysis bullosa acquisita (EBA) is a chronic skin blistering disease of autoimmune origin characterized by antibodies to type VII collagen (COL7)^6^. We previously demonstrated the contribution of MHC haplotype and other non-MHC genes to EBA susceptibility in an immunization-induced model of EBA in mice^7^. Intriguingly, autoimmunity against COL7 is also a common observation among patients with Crohn disease^8^, one of two major forms of inflammatory bowel disease with clear host genetic and microbial components^9^. In this study, we investigate the contribution of host genetic control of the skin microbiota in mice from a four-way autoimmune-prone advanced intercross, enabling loci influencing microbial community structure and disease (EBA) susceptibility to be simultaneously analysed. In addition to identifying host genetic loci that contribute to variability in bacterial taxon abundances in the skin, we find that individual genotype-dependent microbial risk factors modify susceptibility to EBA and increase the power to detect disease-associated loci.

## Results

### Composition and diversity of skin microbiota

**Figure 1:**
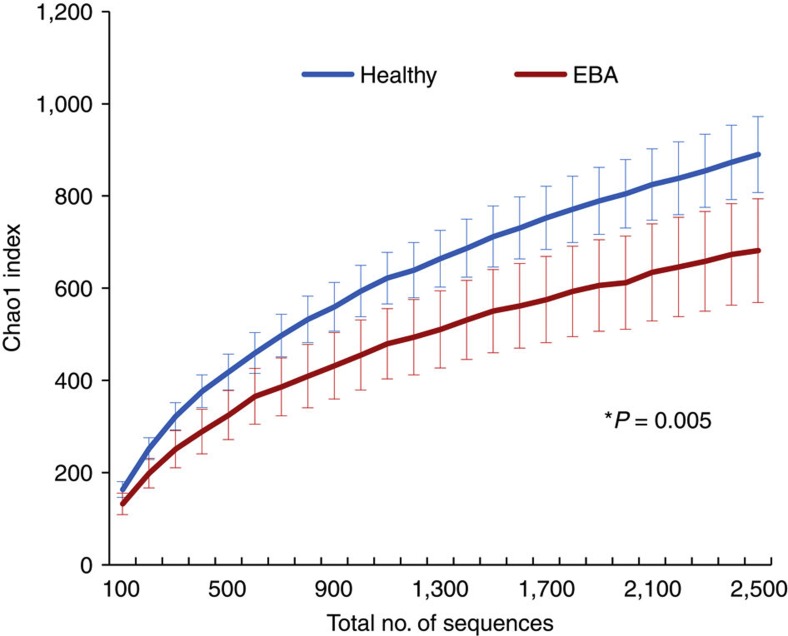
Chao1 species richness. The Chao1 index based on species-level OTUs was estimated for immunized healthy (*n*=119) and EBA (*n*=64) samples. Error bars represent the 95% confidence interval. *Significance was determined by the Wilcoxon rank-sum test.

**Figure 2:**
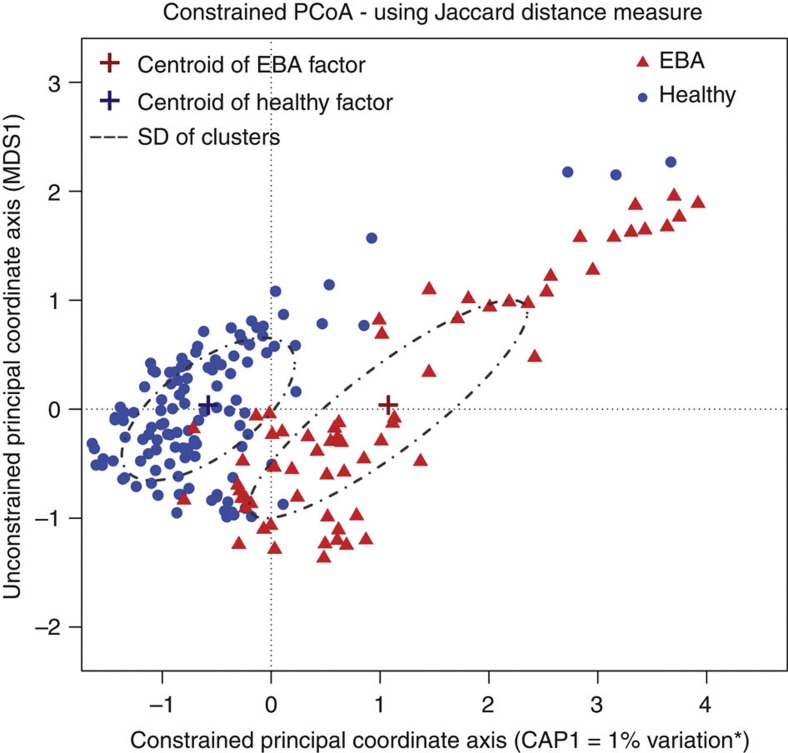
Constrained analysis of principal coordinates of the Jaccard index. The Jaccard index was calculated for immunized healthy (*n*=119) and EBA (*n*=64) samples. The disease status was taken as the constrained factor and differed significantly by permutation test (*P*=0.005, see Methods).

To measure the contribution of host genetics to variation in the mouse skin microbiota, we first analysed 261 individuals from the fourth generation of an advanced intercross lines (183 immunized individuals, of which 64 developed EBA, plus 78 non-immunized controls, Methods) using pyrosequencing of the 16S rRNA gene. At the phyla level, the Firmicutes are most abundant (54%), followed by Proteobacteria (21%), Actinobacteria (12%) and Bacteroidetes (6%) (Supplementary Fig. S1), revealing communities similar to those observed in previous studies of the skin^2^,^10^,^11^,^12^. At the genus level, *Staphylococcus* (36%), *Corynebacterium* (9%) and *Ralstonia* (8%) are most abundant (Supplementary Fig. S2). To characterize the level and pattern of diversity within individuals, we applied different measures of alpha diversity, which focus on species richness, evenness and abundance. The Chao1 species richness index is higher in the healthy individuals compared with those afflicted with EBA (Fig. 1) (Wilcoxon rank-sum test, *W*=170, *P*=0.005). The same pattern is also observed for Faith’s^13^ phylogenetic diversity index (PD whole tree) (Wilcoxon rank-sum test, *W*=176, *P*=0.008), the observed number of species (Wilcoxon rank-sum test, *W*=212, *P*=0.05) and the Shannon evenness measure (Wilcoxon signed-rank Test, *Z*=−4.3726, *P*<0.001) (Supplementary Fig. S3). To analyse bacterial community composition and structure *between* individuals (that is, beta diversity,) we first used the weighted and unweighted Unifrac metric^14^,^15^, which is a phylogenetic-based measure weighted by taxon abundance and based on presence–absence information, respectively. Constrained analysis of principal coordinates (CAP) using EBA status as an explanatory variable and the weighted Unifrac metric as a response variable also reveals a small, but significant effect (*P*=0.015), with the first principal component axis (or CAP1 axis) explaining 1.8% of the variation between individuals (Supplementary Fig. S4a), while the CAP1 axis for unweighted Unifrac explains 2% of the variation (*P*=0.005; Supplementary Fig. S4b). Analysis of beta diversity using OTU-based approaches yields very similar results (Bray–Curtis index, with CAP1 explaining 1.8% of the variation (*P*=0.015) (Supplementary Fig. S4c) and Jaccard index, with CAP1 explaining 1% of the variation (*P*=0.001) (Fig. 2)). For further analysis a ‘core measurable microbiota’ (CMM) of 131 OTUs was defined^3^, which contain nearly 80% of the total sequences in the data set (Supplementary Figs S5 and S6) (Methods).

### QTL analysis of skin microbiota

**Figure 3:**
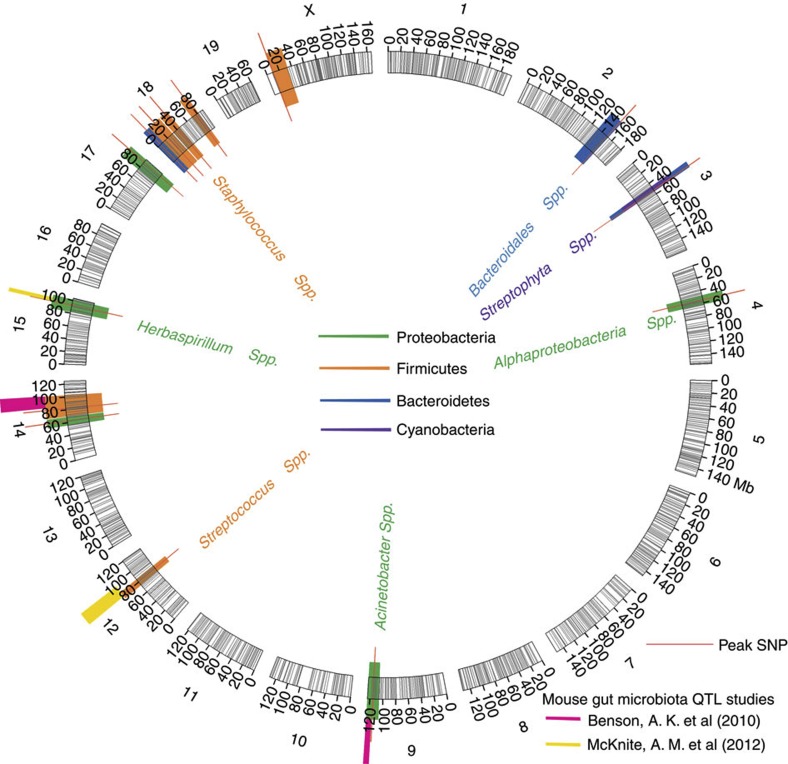
QTL mapping of skin microbiota. Nineteen mouse autosomes and the X chromosome are depicted with 1,199 SNPs indicated by black lines. QTLs for species level OTUs (spQTLs) are colour coded according to their phylum along with their highest reliable taxonomic classification in adjacent text. QTLs from the genus to phylum level (gpQTLs) are colour coded according to their phylum classification (see Supplementary Data 1 and 2).

To account for both environmental and genetic contributions to variation in CMM OTU abundances, a linear mixed model analysis was performed including cage, family and sex as factors (see Methods). This reveals significant cage and family effects accounting for 28% and 3% of the variation in CMM species abundances, respectively. No significant influence of sex is observed for any of the CMM taxa and was thus removed from the model. To measure the genetic contribution, CMM abundances were tested for co-segregation against 1,199 informative single-nucleotide polymorphism (SNP) markers after accounting for cage and family effects. This reveals host genetics to have significant control over members of the skin microbiota, which can be seen in Fig. 3. Nine out of 131 CMM OTUs are associated with three significant (*E-*value cutoff <0.05) and six suggestive (*E-*value cutoff<0.1) (see Methods) species-level OTU QTLs, hereafter referred to as ‘spQTLs’ (Supplementary Data 1). Mapping at higher taxonomic levels including phylum, class, order, family and genus reveals a total of six QTLs, including three with one or more significant associations and three with suggestive associations, hereafter referred to as ‘gpQTLs’ (Supplementary Data 2). Two out of the nine spQTLs are contained within gpQTLs, thus, in total thirteen unique QTLs are identified. To gain further insight we compared our results with previously published QTL studies of the gut microbiota^3^,^4^ and reveal evidence of overlap greater than expected by chance (Fig. 3; Methods). Interestingly, the confidence intervals of our spQTLs and gpQTLs contain five and four genes related to innate immunity, respectively (see Discussion, Supplementary Data 3).

### Effect of immunization and disease status on QTL mapping

As the model of EBA used in this study is immunization-based, we also included 78 non-immunized mice to control for the effect of immunization in the QTL mapping. Accordingly, we analysed a subset where both EBA-afflicted and non-immunized individuals are removed (that is, only the 119 healthy, autoimmunized samples). Despite decreasing the sample size from 261 to 119, two out of nine spQTLs and two out of six gpQTLs are still detected (Supplementary Data 1 and 2). Next, we analysed a subset where the EBA-afflicted mice are removed (that is, including 119 healthy autoimmunized and 78 non-immunized samples). One out of nine spQTLs and two out of six gpQTLs are still detectable despite lowering the sample size from 261 to 197 (Supplementary Data 1 and 2). Thus, the presence of QTLs among subsamples not influenced by differences in disease/autoimmunization status supports the presence of true genetic effects.

### Gene–microbe covariation in disease susceptibility

**Figure 4:**
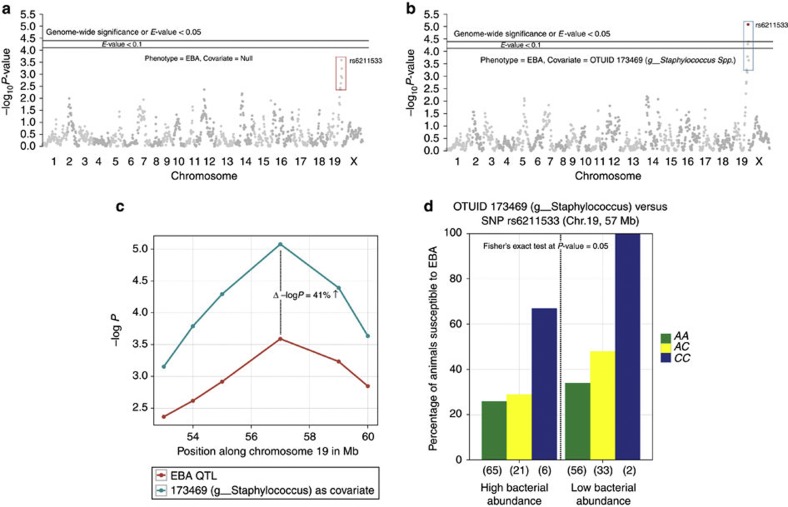
Gene–microbe interaction in EBA susceptibility. (**a**) Manhattan plot of −log_10_ *P-*values for each SNP (1,199 SNPs in *x* axis according to their position on each chromosome) tested against EBA disease phenotype (presence/absence). (**b**) Manhattan plot showing –log_10_ *P*-values for each SNP tested against EBA including *Staphylococcus spp*. (OTUID 173469) abundance as a covariate. SNPs falling below an *E-*value of 0.05 are shown in red. (**c**) Portion of chromosome 19 containing the covariate QTL with a peak at SNP rs6211533. (**d**) Percentage of animals developing EBA among high (top 50%) and low (bottom 50%) *Staphylococcus spp*. (OTUID 173469) abundance categories with respect to host genotype at rs6211533 (represented by green (*AA*), yellow (*AC*) and blue (*CC*)). Numbers in parentheses indicate the sample size within each genotype category.

To investigate the potential role of host genetic variation for skin bacterial abundances in disease, we first re-analysed the subset of 183 immunized mice common to this and our previous study on EBA^7^. This reveals no significant QTL for EBA presence/absence at an *E-*value^16^ cutoff <0.1 (see Methods), likely due to the reduced number of animals (Fig. 4a). However, because bacterial taxon abundance does display a clear genetic component, we next sought to evaluate potential interactions between bacterial species and disease susceptibility by applying a covariate analysis between each of the 131 CMM species abundances and EBA disease susceptibility. This reveals significant covariation (*E-*value <0.1) involving 10 out of 131 taxa, which, intriguingly, increases the power of detecting EBA QTLs, as a novel locus (covariate QTL) (Chr.19, CI 53–60, peak at 57 Mb) is detected (Fig. 4b, Supplementary Data 4). Two OTUs belonging to the genus *Staphylococcus* clearly display a gene–bacterial interaction (*E-*value <0.05) (Fig. 4c).

To further characterize the nature of the identified covariate QTL, we arbitrarily divided individuals into ‘high’ (top 50%) and ‘low’ (bottom 50%) groups with respect to their individual OTU abundances and analysed the proportion of individuals developing EBA with respect to host genotype. This reveals that for most cases the proportion of animals developing EBA is higher among the low OTU abundance category (*n*=10; one of which is also significant by Fisher’s exact test between these defined abundance categories (Fig. 4d); we note, however, that all 10 taxa display significant covariation), suggesting a probiotic role (Supplementary Data 5). Although community-level alterations of the skin microbiota in the context of EBA are present (for example, Supplementary Figs S3 and S4), we note that the putative probiotic covariate taxa identified do not vary in abundance simply according to disease status. Namely, both healthy and diseased individuals are found among the low abundance categories, thus, low abundance of, for example, *Staphylococcus spp*. is not a simple byproduct of disease, but increases the probability of developing symptoms.

**Figure 5:**
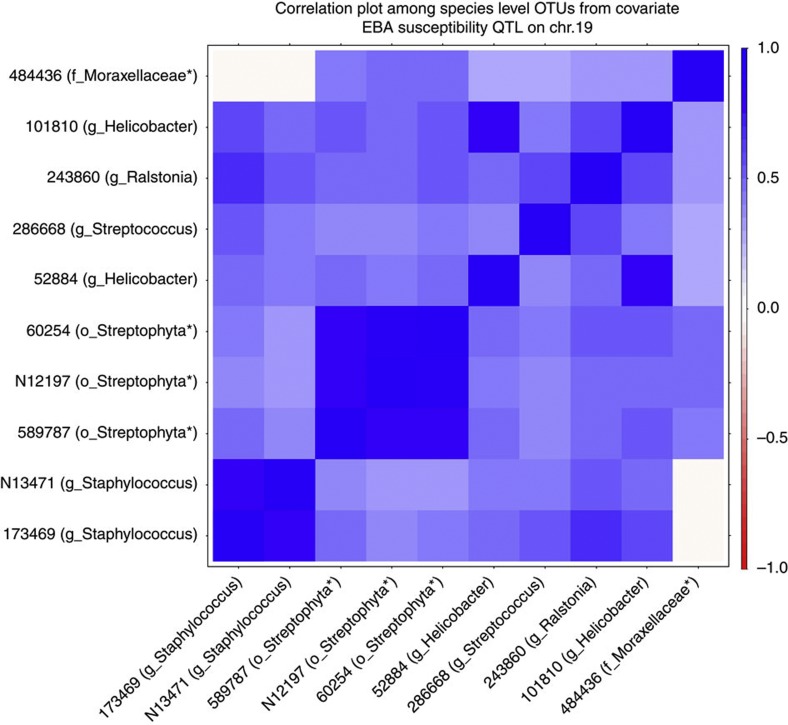
Correlation matrix of covariate OTUs. Pearson’s correlation values between OTUs that significantly co-vary with an EBA susceptibility locus on chromosome 19 are displayed. For OTUs not classified at the genus level, the next highest taxonomic level for which classification was possible is displayed by an asterisk. The taxonomic level of classification is indicated by k, p, c, o, f and g for kingdom, phylum, class, order, family and genus, respectively. Only values significantly differing from zero after correction for multiple testing^17^ are shown by either blue (positive correlation) or red (negative correlation) squares.

The large number of covariate bacterial taxa interacting with a single host locus suggests that individual bacterial taxa may not be acting independently. Thus, to identify potential interactions among covariate taxa we performed a pairwise correlation analysis (Fig. 5). Indeed, this reveals significant positive correlations (Pearson’s correlation; *P-*value ≤0.05 after correction for multiple testing (Benjamini–Hochberg^17^)) between many taxa, suggesting interactions between the host locus and bacterial species assemblages or individual driver species.

## Discussion

Our results provide strong evidence that host genetic variation contributes to differences in the bacterial communities observed in the skin. In a previous QTL analysis of the mouse faecal community, Benson *et al*.^3^ reported 13 significant and 5 additional suggestive QTLs for 26 out of 64 taxonomic groups tested. However, their analysis was not extended beyond the level of bacterial genera. Despite our more inclusive set of phenotypic traits extending to the bacterial species (OTU) level, we detected a smaller number of loci, with nine significant QTLs for 9 out of 131 species-level traits. Differences in sample size, sequencing coverage, mouse strains used and the obvious distinctions between the gut and skin environments may all contribute to these differences in QTL detection. Interestingly, we nonetheless find evidence of overlap between the two studies. The confidence intervals of two out of 18 QTLs controlling bacterial abundance in murine faeces contain the peak SNP of a skin QTL, which overlaps more than expected by chance (*P-*value <0.05; Fig. 3; Methods). One of these QTLs is consistent at the phyla level (Firmicutes, Chromosome 14), while the other is at the order level (*Pseudomonadales*, Chromosome 9). Similarly, an additional two of our skin spQTLs (OTU N31208 belonging to *Streptococcus* on Chromosome 12 and OTU 130241 belonging to *Herbaspirillum* on Chromosome 15) overlap with faecal QTLs from another more recently published study^4^, although the taxonomic assignments do not agree at even the phylum level.

The confidence intervals of our skin microbiota QTLs contain nine genes known to be involved in the functioning of the innate immune system (Supplementary Data 3). *Interleukin-1 receptor-associated kinase (IRAK)-4* is an interesting candidate found within the confidence interval of spQTL6, which modulates an OTU (ID 130241) belonging to the genus *Herbaspirillum*. Deficiencies of this gene in humans lead to increased susceptibility to pyogenic bacterial infections including *Staphylococcus aureus*^18^, and its interaction with the MYD88 adapter protein is used by several Toll-like receptor pathways in host defence^19^, as well as being involved in controlling commensal bacteria^20^. Another gene coding for CD14 antigen is found within spQTL8 on chromosome 18, which modulates an OTU (ID N10459) belonging to the genus *Staphylococcus*. Increasing CD14 expression enhances Toll-like receptor 2 activation in skin in the presence of vitamin D_3_—1,25-dihydroxyvitamin D_3_ (1,25D3)^21^, which in turn influences the skins sensitivity to microbial challenge. Furthermore, several studies have shown that components of *S. aureus* (LTA and peptidoglycan) interact with the CD14 molecule^22^,^23^,^24^. Finally, by treating bacterial abundances as covariates with the presence/absence of EBA, we identified an additional significant EBA QTL on chromosome 19 (Fig. 4). One potential candidate gene lying within this chromosomal interval (53–60 Mb) is *caspase-7* (*casp7*), a member of the cytosolic cysteine protease family known to be involved with inflammatory disorders^25^,^26^ and defence against pathogens^27^.

Similar to previous studies of chronic inflammatory skin diseases, our findings support a role of resident microbial communities in disease pathogenesis. The differences in community composition and structure between mice with and without EBA symptoms are akin to shifts in the skin microbiota associated with atopic dermatitis disease flares and treatment^28^ or between psoriatic lesions and both unaffected skin in patients and healthy controls^29^. Although other examples such as endemic pemphigus foliaceus (fogo selvagum), where exposure to haematophagous insects is implicated^30^, suggest direct environmental/microbial triggers of disease, the roughly three-fold increase over the last 30 years of atopic dermatitis in industrialized countries suggests more complex environmental influences, possibly mediated by changes in microbial communities. By investigating disease provocation in a large mouse mapping population under controlled environmental conditions, we were able to identify individual, genotype-dependent microbial risk factors among a core set of taxa inhabiting the skin of both healthy and diseased mice, more closely resembling a disease-modifying effect. Although further validation and characterization of these interactions awaits more intensive experimental interrogation in, for example, gnotobiotic animals, our investigation of another EBA-susceptible mouse strain (SJL/J; not included in this study) before and after autoimmunization reveals that other aspects of the skin community, in particular alpha diversity (species richness and evenness), are predictive of disease outcome (unpublished results). Thus, the further identification and functional analysis of host genetic and probiotic bacterial factors represent promising avenues for research in preventative and therapeutic treatment development.

## Methods

### Generation of a four-way advanced intercross line

Parental mouse strains (MRL/MpJ, NZM2410/J, BXD2/TyJ, Cast) for generating a heterogeneous intercross line^31^ were purchased from the Jackson laboratory (Maine, USA). Briefly, strains were intercrossed at an equal strain and sex distribution. First generation (G1) offspring mice were then mated considering their parental origin to maintain an equal distribution of parental alleles for successive generations by maintaining at least 50 breeding pairs per generation. Male and female offspring used in the study were transferred to separate cages according to sex after weaning. Depending on the number of animals per cage, females from multiple families were also housed together. Animals were held under specific pathogen-free conditions at a 12-h light/dark cycle with food and water *ad libitum*. All 261 animals (135 males and 126 females) in this study were taken from the fourth generation of this advanced intercross line at 6 months of age. All animal experiments were approved by the state of Mecklenburg-Vorpommern, Germany.

### Induction of experimental EBA

EBA was induced by immunization with an immunodominant peptide within the murine NC1 domain of type VII collagen (GST-mCOL7C)^7^. In brief, 60 μg GST-mCOL7C emulsified in 60 μl adjuvant (TiterMax, Alexix, Lörrach, Germany) was injected subcutaneously into the foot pad and tail base. After immunization mice were screened for skin inflammation every 4th week for a period of 12 weeks, after which the ears were taken for analysis at 6 months of age. Ear skin samples were fixed in 4% buffered formalin and snap frozen at −80 °C. A total of 183 immunized- and 78 non-immunized mice were included in skin microbiota QTL mapping.

### DNA extraction and 16S rRNA gene pyrosequencing

Bacterial DNA from mouse ears was extracted using the PowerSoil Kit (MoBio, Carlsbad, CA). During the killing of the mice, both ears were taken for either bacterial DNA extraction or other analyses (that is, histology, RNA), and the ear chosen for microbial analysis was chosen at random. Approximately one third of an ear was transferred to the Power Bead tubes containing 60 μl of C1 solution and 20 μl of 20 mg ml^−1^ Proteinase K. Samples were incubated at 50 °C for 2 h at 850 r.p.m. and the remaining steps were performed according to the manufacturer. Amplification of the hypervariable V1 and V2 (27F–338R) region of the 16S rRNA gene was performed using composite forward (5′-*CTATGCGCCTTGCCAGCCCGC*TCAGTCAGAGTTTGATCCTGGCTCAG-3′) and reverse (5′-*CGTATCGCCTCCCTCGCGCCA*TCAGXXXXXXXXXXCATGCTGCCTCCCGTAGGAGT-3′) primers. These primers include the 454 Life Sciences adaptor A (reverse) and B (forward), denoted by italics. The underlined sequences represent the broadly conserved bacterial primers 27F and 338R. Ten base pair multiplex identifiers (MIDs; designated as “XXXXXXXXXX”) were added to reverse primers to uniquely tag PCR products. Duplicate 25 μl PCR reactions, each containing 100 ng of template DNA, were performed using Phusion Hot Start DNA Polymerase (Finnzymes, Espoo, Finland) with the following cycling conditions: initial denaturation for 30 s at 98 °C; 35 cycles of 9 s at 98 °C, 30 s at 55 °C and 30 s at 72 °C; final extension for 10 min at 72 °C. Duplicate reactions were combined after PCR and products were extracted with the MiniElute Gel Extraction Kit (Qiagen, Hilden, Germany). Quantification was performed with the Quant-iT dsDNA BR Assay Kit on a Qubit fluorometer (Invitrogen, Darmstadt, Germany). Purified PCR products were pooled in equimolar amounts and further purified using Agencourt Ampure Beads (Beckman Coulter, Krefeld, Germany). Aliquots of each library were run on an Agilent Bioanalyser before emulsion PCR and sequencing according to the manufacturer’s instructions on a Roche 454 GS-FLX using Titanium sequencing chemistry.

### 454 Pyrosequencing data analysis

A Perl script using the Smith–Waterman algorithm^32^ was written to match the forward primer and barcode allowing no insertions or deletions. Sequences were required to have a length between 290 to 370 nucleotides, an average quality score ≥20 and contain no ambiguous bases. ChimeraSlayer (http://microbiomeutil.sourceforge.net/) was used to remove chimeras. An average of 5,732 reads per sample was obtained for 261 animals.

### Taxonomic classification

RDP classifier^33^ (RDP Multi-Classifier version 1.0) was applied to assign taxonomy to the genus level using 0.80 as minimum confidence. The ‘fixrank’ option was used to assign taxonomy from kingdom to genus level. QIIME^34^ scripts were used to pick OTUs at a 97% similarity threshold. The most abundant sequence from each OTU bin was chosen as the representative sequence. Species-level taxonomy was obtained by NCBI BLAST against the greengenes reference database (http://greengenes.lbl.gov/Download/Sequence_Data/Fasta_data_files/Caporaso_Reference_OTUs/gg_otus_4feb2011.tgz) with an *E*-value cutoff of 0.001. Sequences unclassifiable at a given taxonomic rank were classified at the next possible higher rank.

### Data preparation for QTL analysis

The proportion values (number of reads for a given taxon/total sequence reads for a given animal) for each taxonomic level were normalized as suggested by Benson *et al*.^3^ The taxon bins were then log_10_ transformed. This resulted in 34,624 species-level OTUs, 863 genera, 376 families, 218 orders, 93 classes and 51 phyla. Out of 34,624 species-level OTUs, 19,443 cover nearly 99% of the total sequences and 762 species OTUs represents nearly 90% of the total sequences (Supplementary Fig. S6).

### Core measurable microbiota

A CMM was determined by a manner similar to that of Benson *et al*.^3^ using two technical repeats from five different samples. Sequences were processed, classified into taxonomic bins and the log_10_ transformed values for each bin were plotted for all pairwise combinations of the two repeats (Supplementary Fig. S5). A threshold of >20 reads per bin leads to a correlation >0.97. Thus, the CMM taxa were defined as bins containing more than 20 reads in at least 20 animals. The resulting 131 species-level OTUs represent nearly 80% of the total sequences (Supplementary Fig. S6).

### Genotyping and QTL analysis

The Illumina murine HD array was used to genotype 1,449 SNPs (of which 1,199 are informative) from 261 G4 animals. The Happy package^35^ was used for QTL and covariate QTL analysis, expecting additive contributions from inherited parental haplotypes at each locus. Each log_10_ transformed taxonomic bin was treated as an individual phenotypic trait. The hfit function in Happy was used to correct for cage and family effects in a manner similar to Johnsen *et al*.^36^ Covariates were included in the model by specifying an additional design matrix in the input file. The genome-wide significance, or *E-*value, for each phenotype was estimated by a permutation test based on 1,000 shuffled reassignments of the phenotypes^16^. To illustrate the procedure used to obtain the *E-*value for a given trait, we produced a ‘QQ-plot’ (Supplementary Fig. S7), which shows the distribution of maximum −log_10_ *P-*scores recorded across 1,199 SNPs for 1,000 random permutations of the phenotype scores (for OTUID: N26684). The threshold for significant QTLs was set at 5% and that for suggestive QTLs^3^ at 10%. This corresponds to analysis of variance (ANOVA) −log_10_ *P-*values ≥4.39 and ≥4.10, respectively, as described by Valdar *et al*.^37^ Confidence intervals were determined manually by a drop of 1.5 in ANOVA −log_10_ *P*-score^16^. The probability of overlap for QTLs was calculated as described by Graham *et al*.^38^ with a size of 2,500 Mb used as the size of the mouse genome. Chromosome visualization was performed with circus^39^. All mouse genotype, phenotype and Happy input files are provided in Supplementary Data 6.

### Alpha and beta diversity analysis

Alpha and beta diversity analyses were largely performed using QIIME^34^. Alpha diversity^34^,^40^ analysis was performed on the whole data set with a normalized sequence number of 2,500 per individual (the lowest read number in the data set). An OTU table was generated using a 97% sequence similarity threshold and singletons were removed. The Chao1 and Shannon indices, both common measures of diversity within a sample (that is, alpha diversity), were used to describe species richness and evenness, respectively. Between-sample diversity (that is, beta diversity) was analysed using CAP implemented in the Vegan package in R^41^, with disease status as a constraint. This analysis is very similar to a redundancy analysis, but additionally it allows non-Euclidian dissimilarity indices (we used Bray–Curtis^42^, Jaccard and UniFrac^14^,^15^ distances). Statistical significance for CAP was determined by an ANOVA-like permutation test function with 1,000 permutations (anova.cca) in Vegan. We used R version 2.15.2 on Linux (*R* Development Core Team (2012)).

## Author contributions

G.S. and S.K. performed experiments, G.S., S.M. and J.W. performed analysis; G.S., J.F.B. and S.M.I. wrote the paper; J.F.B., S.M.I., and D.Z. edited the paper and assisted in interpretation; J.F.B. and S.M.I. designed the experiments.

## Additional information

**Accession code:** European Nucleotide Archive (ENA). ERP002614. PRJEB1934 (http://www.ebi.ac.uk/ena/data/view/PRJEB1934)

**How to cite this article:** Srinivas, G. *et al*. Genome-wide mapping of gene–microbiota interactions in susceptibility to autoimmune skin blistering. *Nat. Commun.* 4:2462 doi: 10.1038/ncomms3462 (2013).

## Supplementary Material

Supplementary Figures Supplementary Figures S1-S7

Supplementary Dataset 1 QTLs detected for species level OTUs of the skin microbiota

Supplementary Dataset 2 Skin microbiota QTLs detected from genus to phylum level

Supplementary Dataset 3 List of innate immunity related genes found within the confidence intervals of spQTLs

Supplementary Dataset 4 Covariate analysis using EBA susceptibility as the primary phenotype and bacterial species OTUs as covariates

Supplementary Dataset 5 Percentages of animals developing EBA with respect to genotype and bacterial abundance class (high or low)

Supplementary Dataset 6 All mouse genotype, phenotype and Happy input files
