# Supplementary material for: Genome-wide mapping of gene–microbiota interactions in susceptibility to autoimmune skin blistering
Source: Nat Commun. 2013 Sep 17;4:2462. doi: 10.1038/ncomms3462 (PMC3778513; doi:10.1038/ncomms3462)
Supplement: Supplementary Figures — S1-S7 [file ncomms3462-s1.pdf]

a

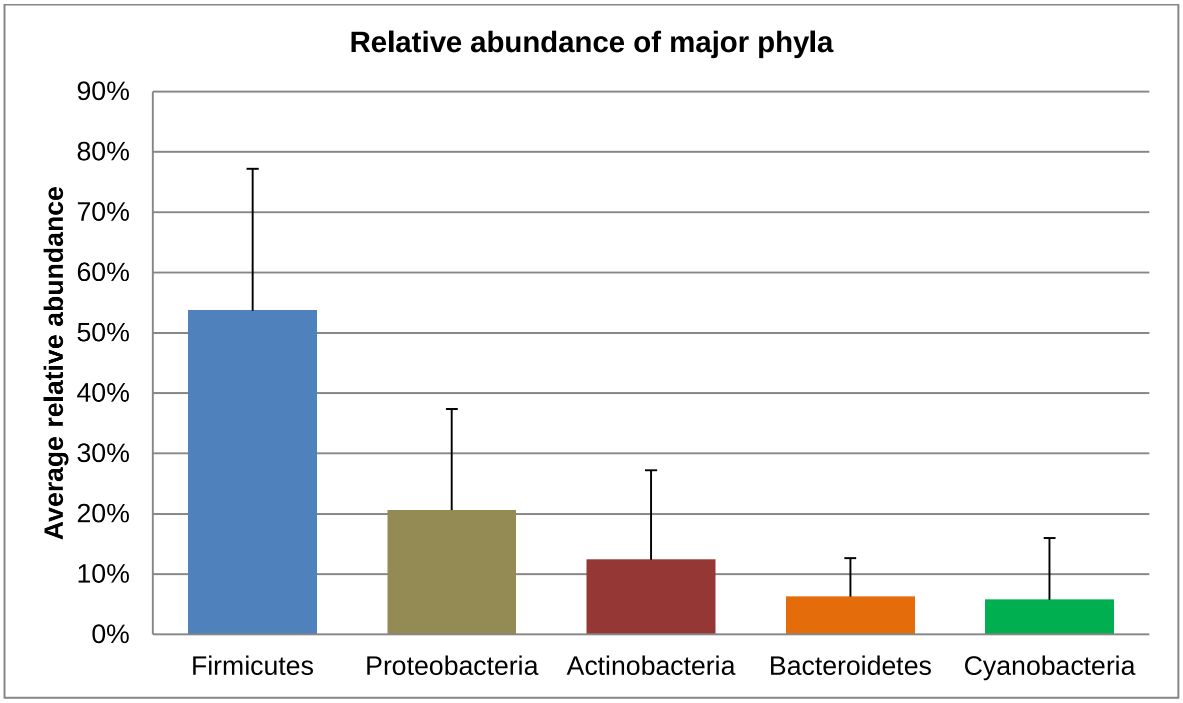

b

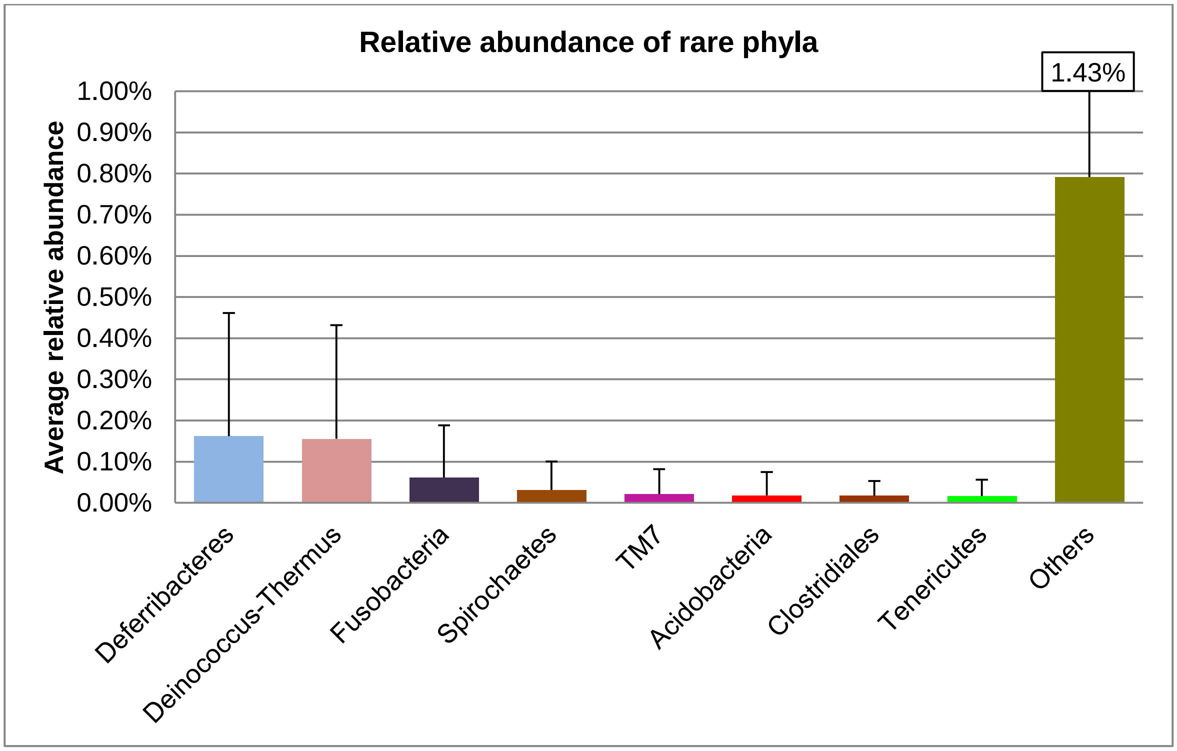

Supplementary Figure S1: **Skin bacterial composition at the phylum level.**
(a) Relative abundance of major and **(b)** minor phyla of the mouse skin microbiota (n=261). Error bars indicate 1 standard deviation (SD).

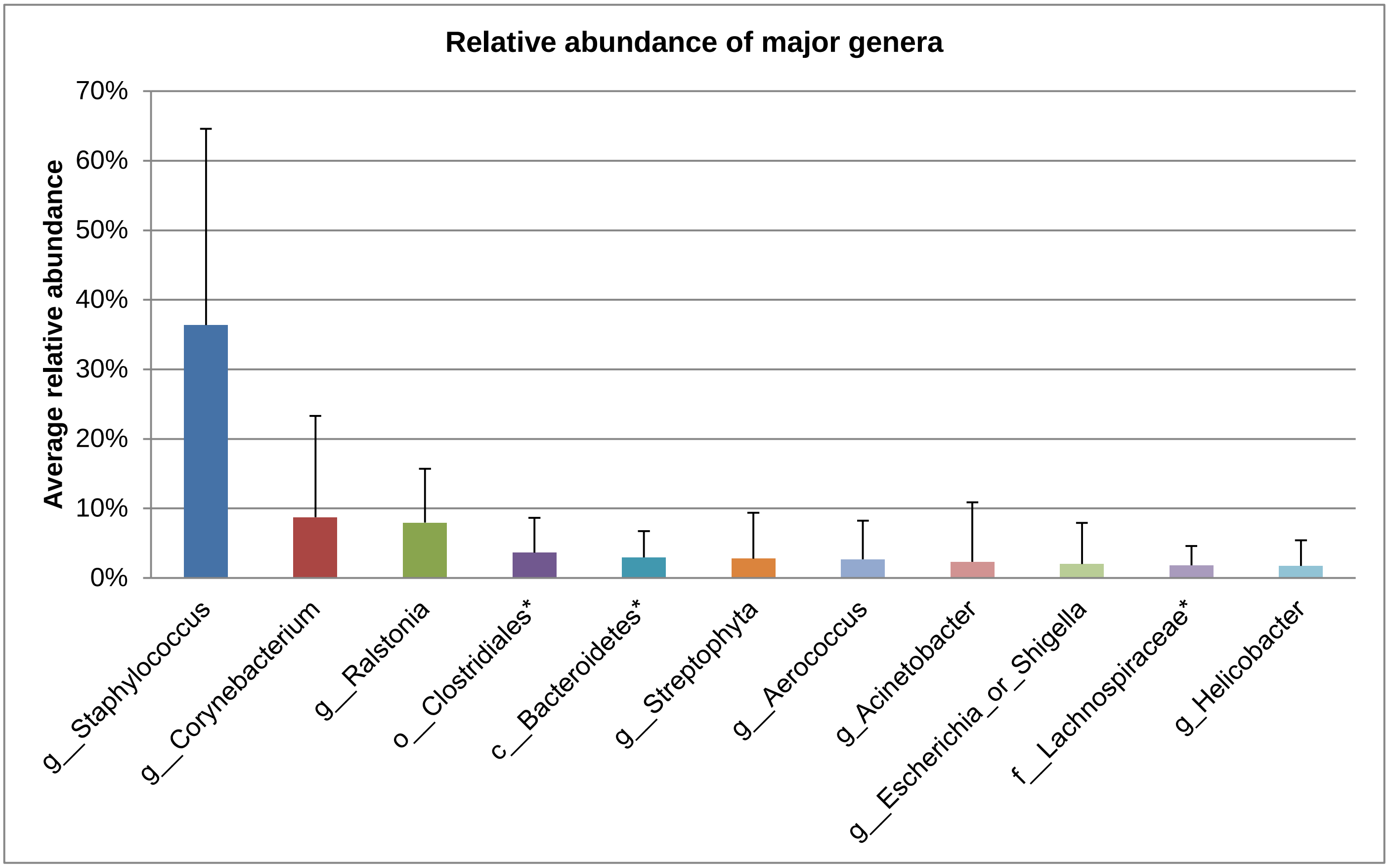

Supplementary Figure S2: Skin bacterial composition at the genus level.
Ten most abundant genera in the mouse skin microbiota (n=261). For those unclassified at the genus level, the next highest taxonomic level for which classification was possible is displayed by an asterisk. The taxonomic level of classification is indicated by k, p, c, o, f and g for kingdom, phylum, class, order, family and genus, respectively. Error bars indicate 1 SD.

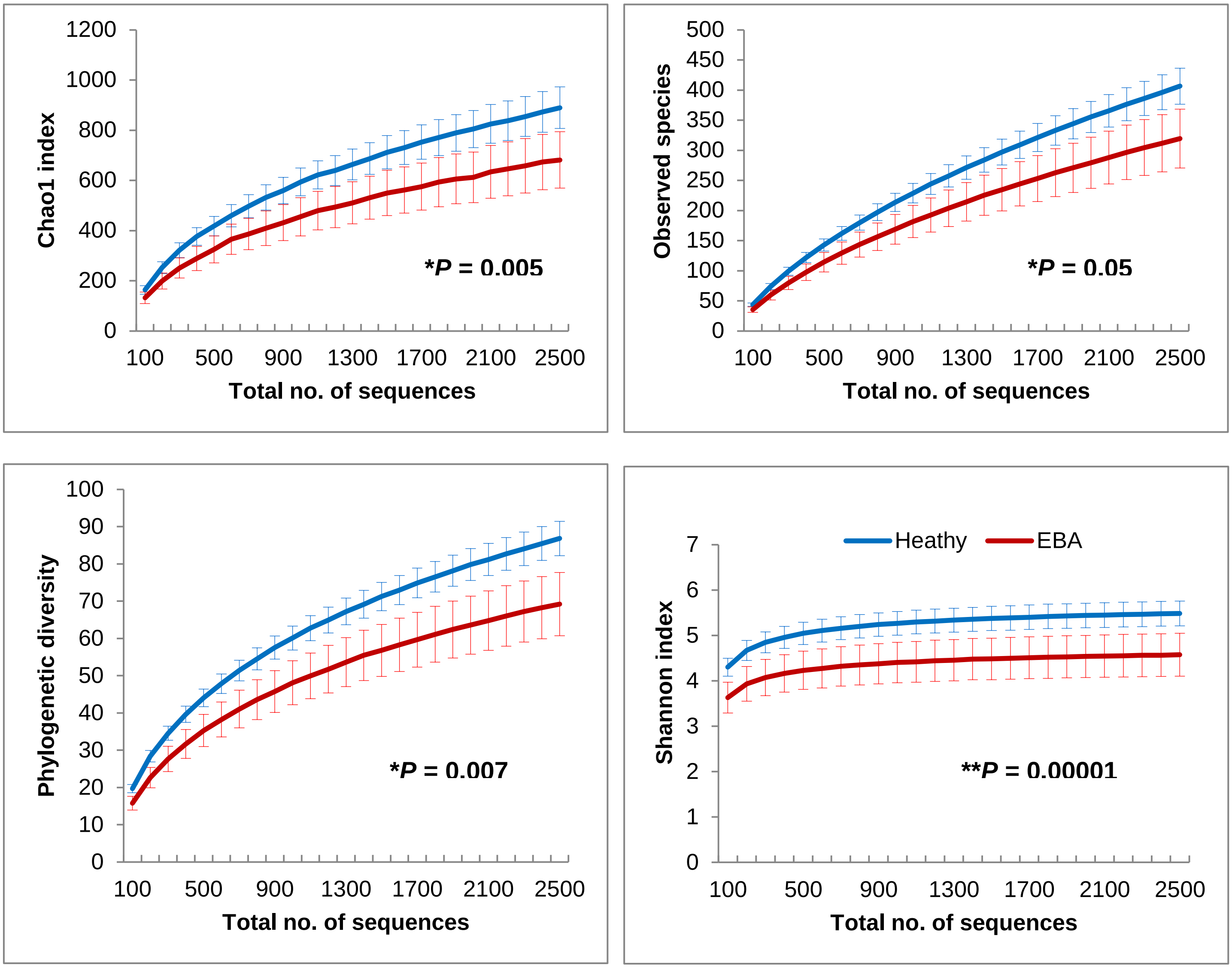

Supplementary Figure S3: Rarefaction curves of alpha diversity indices for species level OTUs
The indices are calculated for immunized healthy (n=119) and EBA (n=64) samples. Error bars represent the 95% confidence interval.*Significance was determined by the Wilcoxon rank sum test in R.
**Significance was determined by the Wilcoxon signed rank test.

a

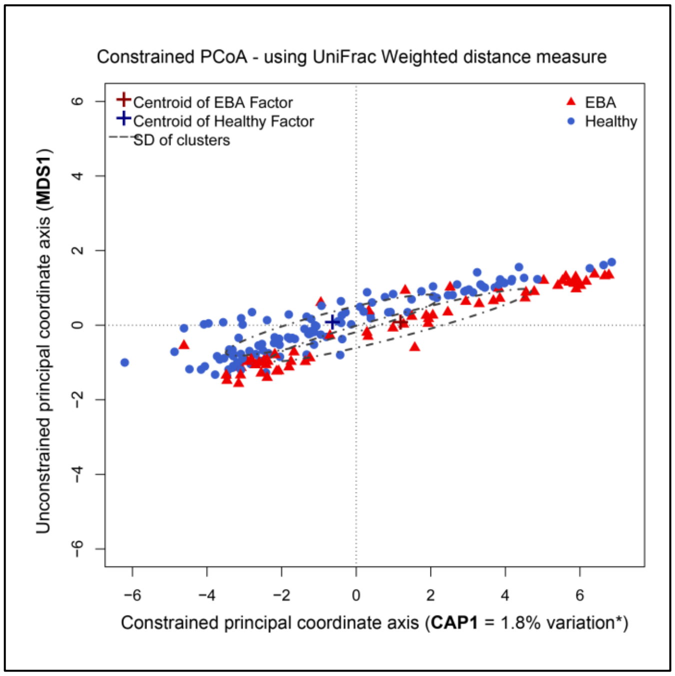

b

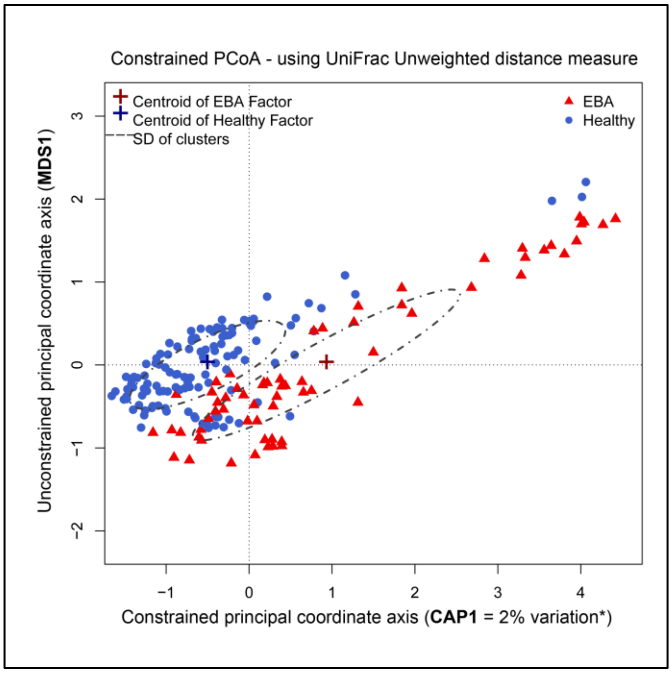

c

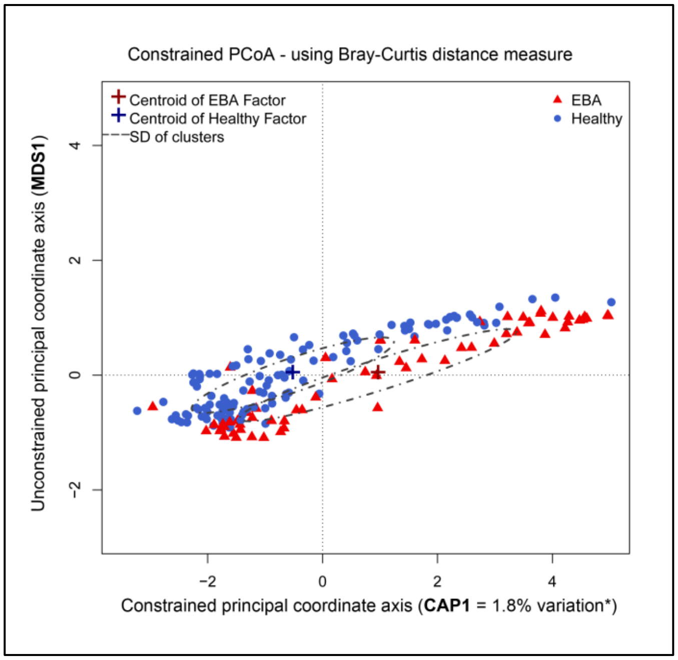

Supplementary Figure S4: Constrained Analysis of Principal Coordinates analysis of beta diversity indices.

Beta diversity indices were calculated among immunized healthy (n=119) and EBA (n=64) samples. The disease status was taken as the constrained factor and significance was determined by permutation test. (a) Weighted UniFrac metric *P = 0.015 , (b) unweighted UniFrac metric *P = 0.005, and (c) Bray-Curtis index *P = 0.015.

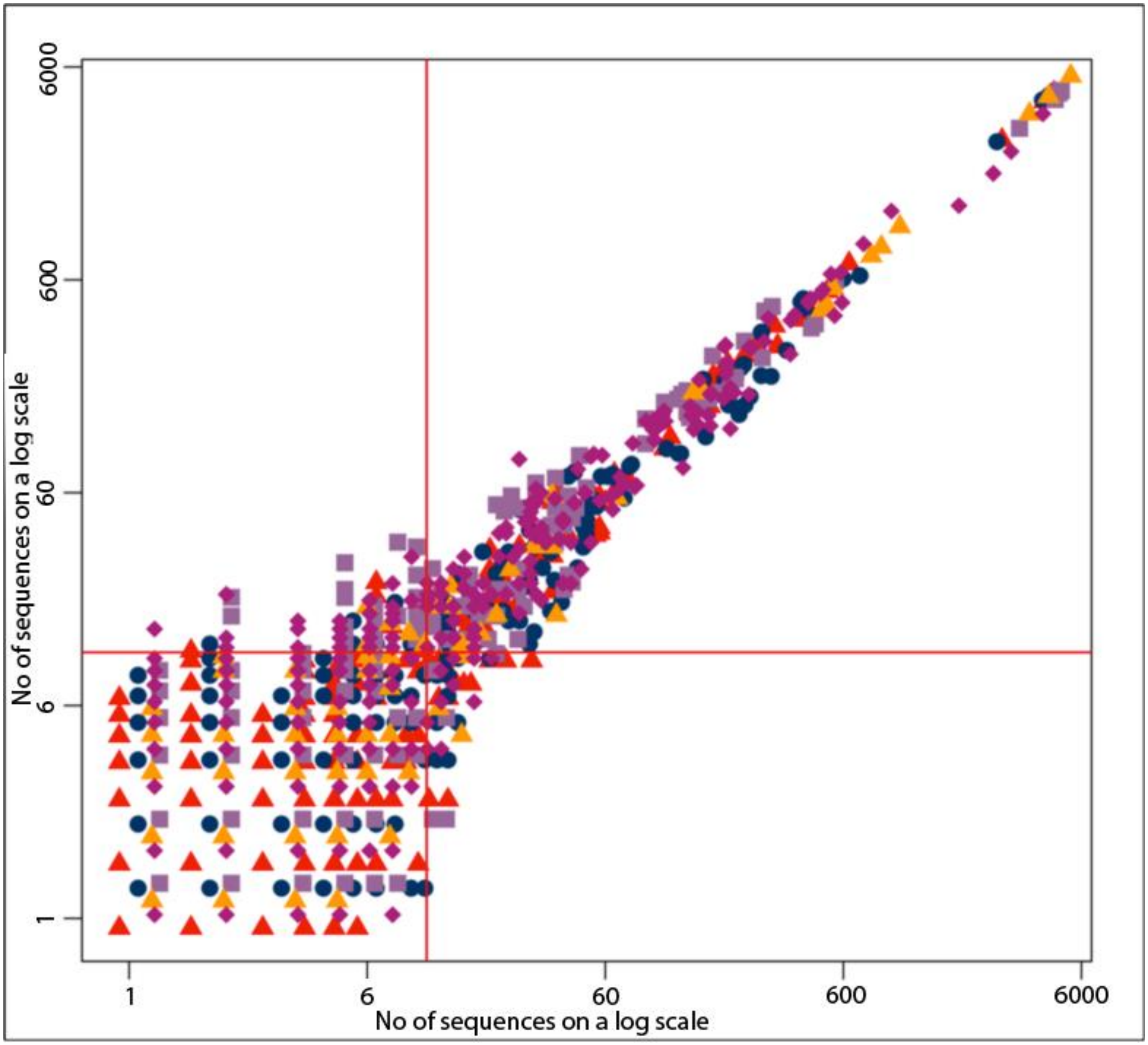

Supplementary Figure S5: Scatterplot of technical repeats performed on five different samples.

Pairwise comparisons of taxonomic bins between technical replicates for each sample are displayed by a different color/symbol per sample. The V1-V2 region of the 16s rRNA gene from each sample was amplified with two different sets of barcoded primers. The sequence data was processed and taxonomic assignments were performed (see **Methods**). Sequence counts for each taxonomic bin were log-transformed and plotted for all pairwise comparisons of the two technical repeats for each sample. Taxonomic bins falling above the red lines indicate those with a correlation >0.97 between replicates and correspond to having at least 20 reads per bin. Thus, OTUs with at least 20 reads per bin and occurring in at least 20 animals were chosen to comprise the “Core Measurable Microbiota” (CMM).

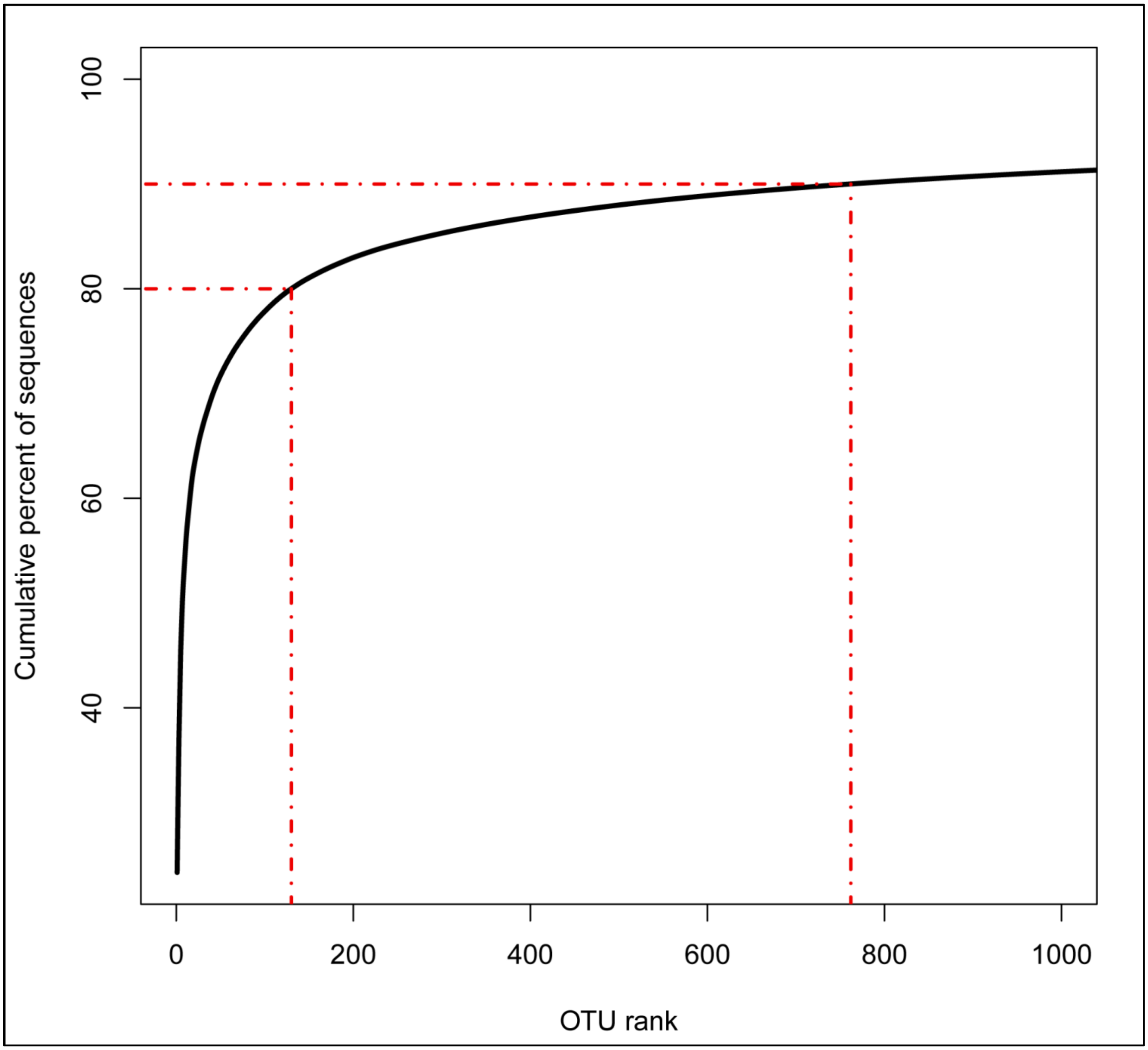

Supplementary Figure S6: Distribution of sequences among the 1000 most abundant OTUs.

The X-axis indicates the individual species level OTUs ranked according to their relative abundance from high to low. The Y-axis indicates the cumulative percent of the total number of sequences. The dotted lines represent 80% and 90% of the total sequence reads from 261 samples, corresponding to the 131 and 762 most abundant OTUs, respectively.

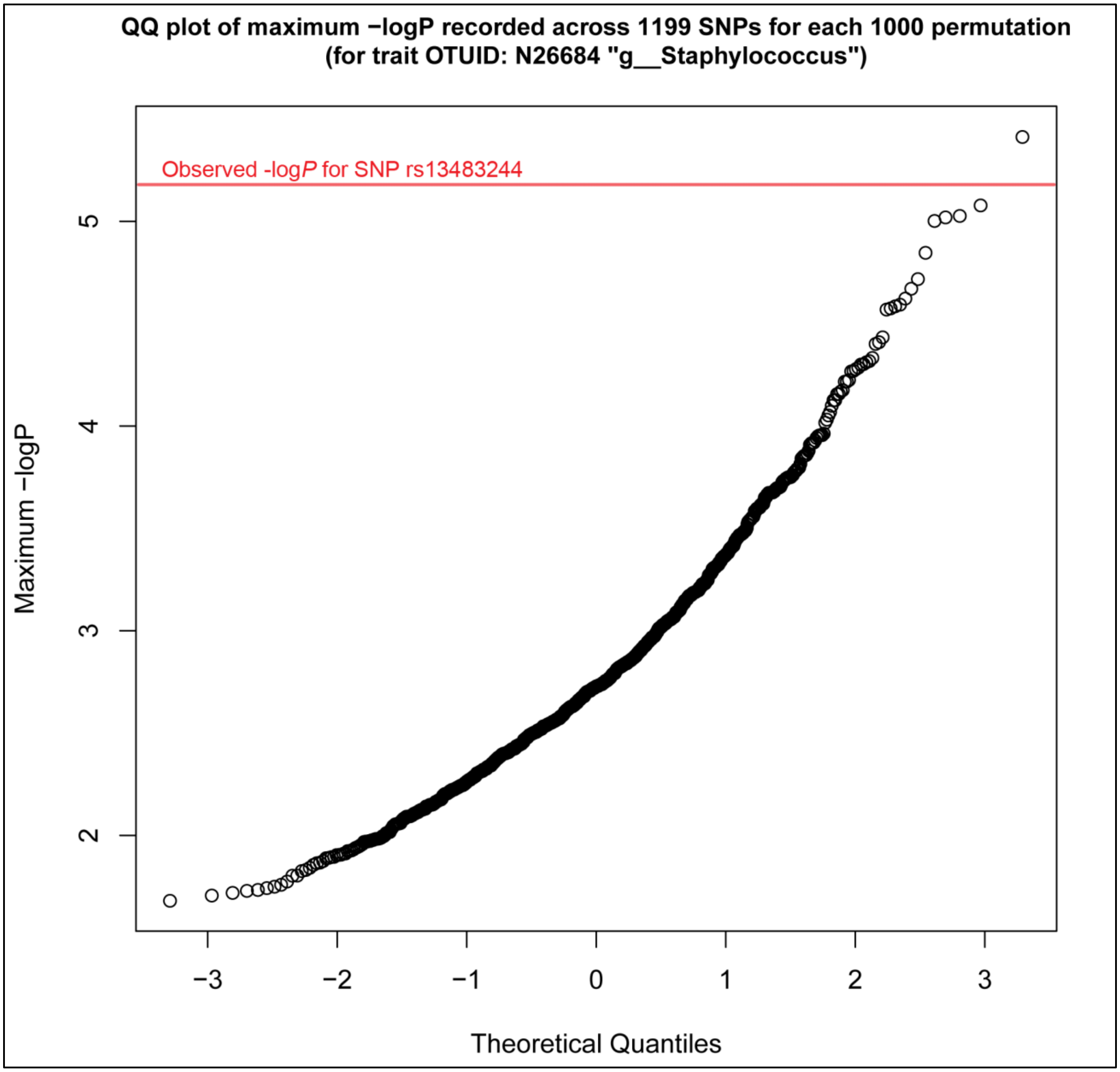

Supplementary Figure S7: Maximum –log*P* scores across 1199 SNPs for 1000 random permutations of the phenotype scores for OTUID N26684

This analysis (a.k.a. "QQ plot") was used to estimate the genome-wide significance threshold, or "*E* value" (see ref. 38 in the main text). For example, the *E* value for SNP rs13483244 would be 1/1000 from the above plot (observed –log*P* value shown in red for trait OTUID: N26684 "g_Staphylococcus"). In other words, the probability of the observed –log*P* score for SNP rs13483244 (which is 5.24) to occur by chance is 1 in 1000.
